# Optical Indoor Positioning System Based on TFT Technology

**DOI:** 10.3390/s16010019

**Published:** 2015-12-24

**Authors:** István Gőzse

**Affiliations:** Institute for Computer Science and Control, Hungarian Academy of Science, Kende St. 13–17, 1111 Budapest, Hungary; gozse@sztaki.hu; Tel.: +36-1-279-6000 (ext. 6148); Fax: +36-1-466-7483

**Keywords:** optical sensors, optical indoor positioning, TFT technology

## Abstract

A novel indoor positioning system is presented in the paper. Similarly to the camera-based solutions, it is based on visual detection, but it conceptually differs from the classical approaches. First, the objects are marked by LEDs, and second, a special sensing unit is applied, instead of a camera, to track the motion of the markers. This sensing unit realizes a modified pinhole camera model, where the light-sensing area is fixed and consists of a small number of sensing elements (photodiodes), and it is the hole that can be moved. The markers are tracked by controlling the motion of the hole, such that the light of the LEDs always hits the photodiodes. The proposed concept has several advantages: Apart from its low computational demands, it is insensitive to the disturbing ambient light. Moreover, as every component of the system can be realized by simple and inexpensive elements, the overall cost of the system can be kept low.

## 1. Introduction

A positioning system is an architecture of sensors, processing units and software components designed to determine the position coordinates of moving objects (*i.e.*, people, devices or vehicles). The most widely-used positioning system is the global navigation satellite system (GNSS), which works well in most outdoor applications (vehicle navigation, routing, *etc.*), where a general and global solution is needed. Indoor positioning systems come into the picture when the objects have to be localized in such environments where GNSS performs poorly. These environments are mostly restricted, closed areas, typically inside buildings, where several factors make the use of GNSS challenging or even impossible: Non-line-of-sight conditions, signal reflections from walls, a greater density of obstacles and special requirements (*i.e.*, demand for higher precision and accuracy, *etc.*). On the other hand, there are further factors that facilitate the indoor positioning and can be exploited to improve the precision and accuracy: Small coverage areas, low weather influences and the availability of infrastructure, such as electricity and the Internet. Due to all of these special features, indoor positioning is not dominated by one technology, as outdoor positioning is by satellite-based location services. Thus, for indoor positioning, a large number of basically different solutions exist [1,2,3]. In this paper, a novel technology is proposed that differs, to our knowledge, from all other technologies used for indoor positioning so far. The main application fields we are targeting are industrial automatized production, motion capture and mobile-robot tracking, *i.e.*, where multiple object detections of high precision and high speed tracking are required. In the sequel, a short review on the indoor positioning systems is given.

Camera-based systems use camera images to determine the position of the markers mounted on the objects [4]. The position of the marker on the image determines the angle subtended by the line connecting the camera to the object (line-of-sight) and the center line of the camera. The position is calculated from multiple, simultaneous angle measurements made from different locations. Camera-based positioning systems can be classified into three categories. Passive marker systems use markers that reflect the ambient or artificially-emitted light. For example, the Vicon system, which is well known from autonomous robot applications [5], is based on this technology. The next group, formed by the active marker systems, uses light-emitting markers. Active systems have advantages over passive ones, because the active markers can be identified more easily, and the system is less sensitive to marker swapping. On the other hand, the active markers are more expensive. Finally, the third group of camera-based positioning systems is formed by markerless solutions, where the objects are identified on the basis of their particular features, so that there are no dedicated markers mounted on them.

It is common in all camera-based solutions that the reliable recognition of the markers requires complex image processing algorithms, which require high computational power, especially in the case of high speed applications. This can significantly increase the cost of the positioning system. Moreover, the camera-based systems are sensitive to ambient light; they easily lose their reliability if the light coming from the environment is rapidly changing, *i.e.*, the bandwidth of the auto gain control of the brightness is lower than the rate of change.

An architecture with three orthogonal photodiodes is used in [6,7]. Many indoor positioning systems are based on measuring the propagation time of artificially-emitted sound [8,9,10]. Magnetic field measurement is another possible method to determine position [11,12]. A large group of indoor positioning systems uses infrared light [13,14].

The proposed positioning system is similar to the active marker camera-based systems. The objects are marked with LEDs (light-emitting diodes), and there are sensor units that act like a camera, measuring the angle of arrival of the light. The difference is that the marker recognition and position measurement is realized by special sensor units. Hence, the positioning system consists of a number of individual sensor units that are mounted at fixed positions, so that each segment of the space of interest is in the field of vision of at least two units. The individual data of the markers are collected from the sensor units, and the position of a marker is calculated. The main advantage of the technique, apart from its low computational demands, is its insensitivity to the disturbing ambient light. In addition, it can be constructed from inexpensive elements [4].

The paper is organized as follows. Section 2 discusses the basic operational principles of the proposed system. The light sensors are described in Section 3. The results of Section 2 and Section 3 have been published in [15]. For the sake of completeness, these sections contain revised explanations of the results. Section 4 is dedicated to the mathematical model of the positioning system. Section 5 elaborates the control algorithm related to the sensor unit. Section 6 discusses the prototype and presents some experimental results. The last section provides final conclusions and directions for future work.

## 2. Operation Principle

**Figure 1:**
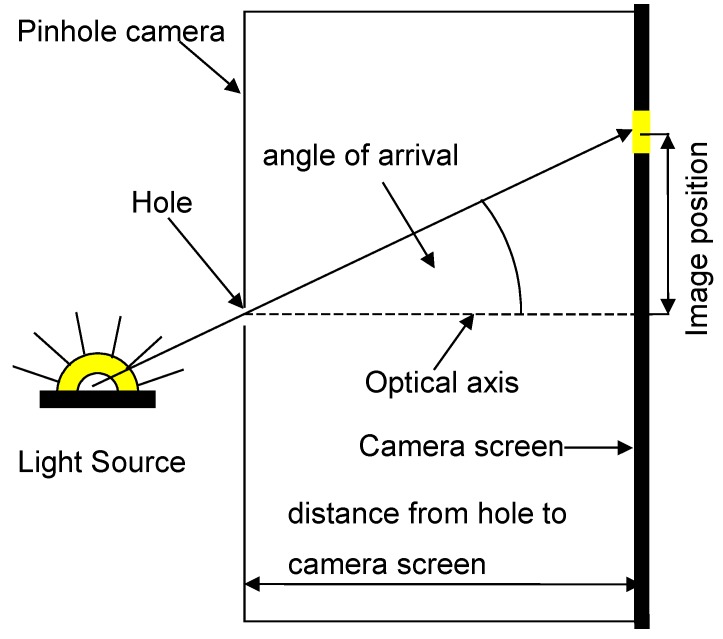
Angle of arrival determination with a simple pinhole camera.

**Figure 2:**
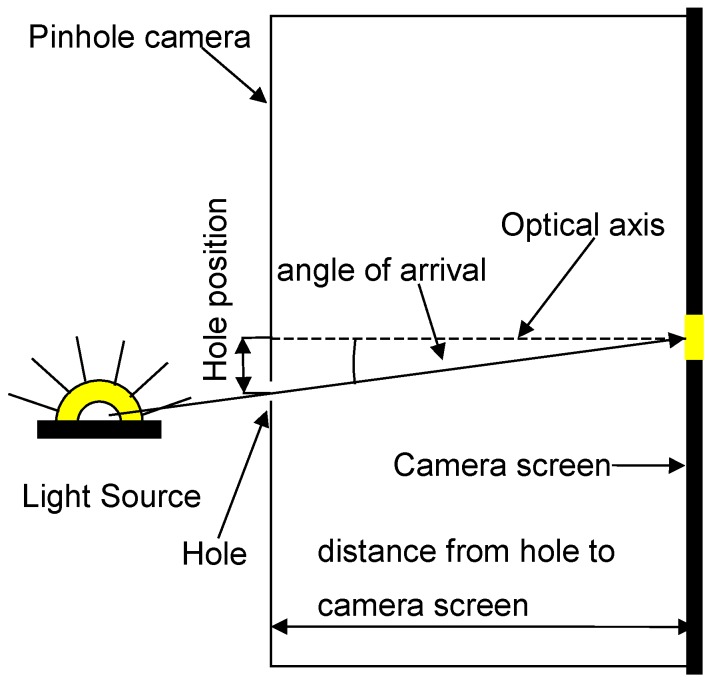
Angle of arrival determination with a modified movable hole pinhole camera.

To explain the main idea behind the proposed method, we recall first the main concept of camera-based positioning. For this, consider a pinhole camera model and a point light source moving in front of the camera (see Figure 1). The direction of the light source relative to the camera can be characterized by the angle of arrival, which is the angle between the optical axis and the line connecting the light source with its 2D image on the camera screen (see Figure 1). The angle of arrival can be determined from the position of the image and the distance between the screen and the hole. In this setup, the position of the hole is fixed, while the location of sensing (*i.e.*, the position of the image on the screen) changes according to the actual position of the light source. Now, let the roles be flipped: let the location of sensing be fixed at the center of the screen, and allow the hole to move. Then, the angle of arrival can be determined as follows: Move the hole until the sensor can see the light source, then compute the angle by using the position of the hole and the distance between the hole and the screen (see Figure 2). Therefore, the algorithm is as simple as in the previous case. At the same time, this slight conceptual modification has a significant advantage: a pinhole camera with a movable hole can be realized with simple elements, which results in a significant reduction in the cost and complexity of the positioning system.

**Figure 3:**
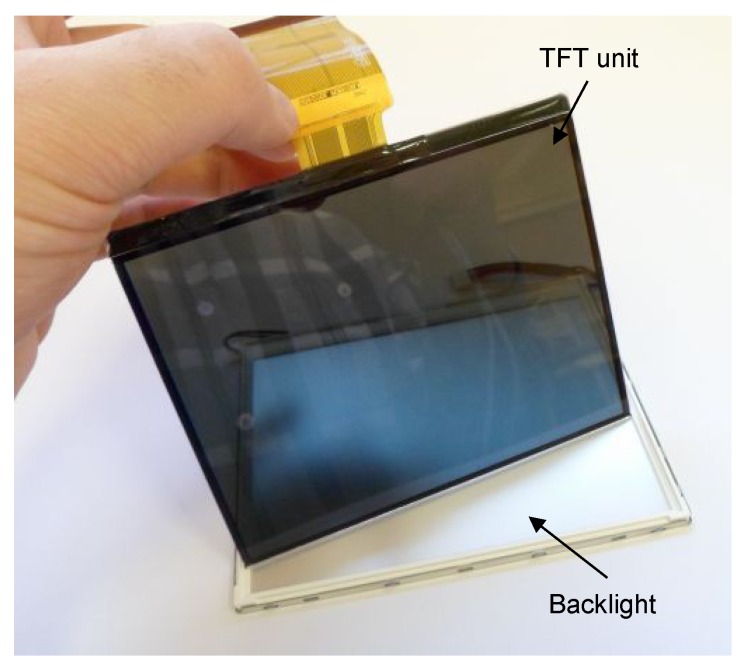
Disassembled TFT module.

The front panel of the pinhole camera can be constructed from a TFT (thin film transistor) unit. A regular TFT display can be disassembled into a TFT unit and a back light panel, as shown in Figure 3. If one uses only black and white colors in the image plane, the TFT unit can be interpreted as a special window that can be transparent or opaque according to the image displayed on it. Thus, by leaving only a small transparent patch and driving every other pixel of the TFT unit into the opaque state, a hole can be realized. This hole is moveable, as well as the patch can be placed anywhere on the TFT. 

**Figure 4:**
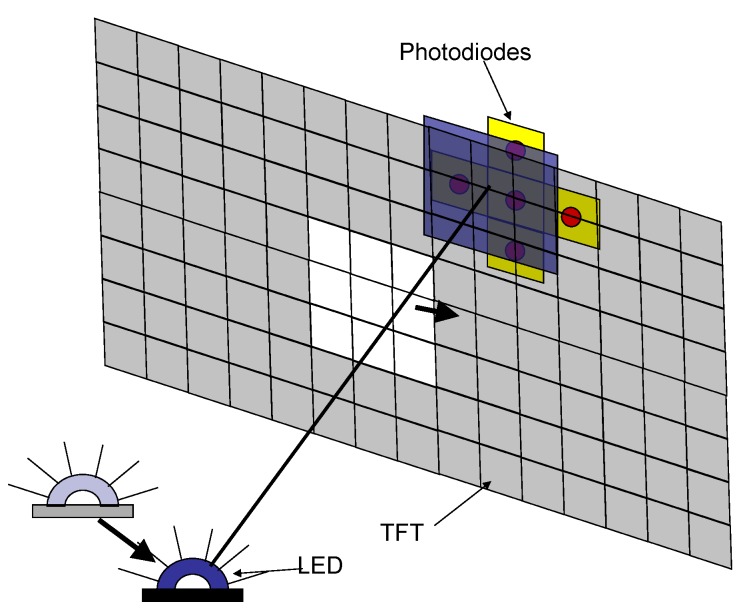
The LED tracking.

The point light source can be a light-emitting diode (LED), while the detector, which is placed in the center of the image plane behind the TFT unit, can be any light-sensitive element, e.g., a photodiode. When the patch tracks the LED (marker), the patch is moved in a way that the light of the LED always hits the photodiode. The instrument can maintain continuous direction measurements frame by frame that are displayed on the TFT panel. To simplify the marker tracking task, four independent photodiodes are used according to Figure 4.

The photodiodes form a square-shaped array where a photodiode is placed on each corner of the square. This sensor array is rotated 45 degrees relative to the TFT pixel grid. This arrangement allows one to determine the heading of the marker. The fifth photodiode in the middle of the sensor array measures the absolute value of the luminous intensity of the LED. Multiple marker detection is also possible, because on the same TFT image, a multiple-hole pinhole camera can be created by using multiple patches simultaneously.

**Figure 5:**
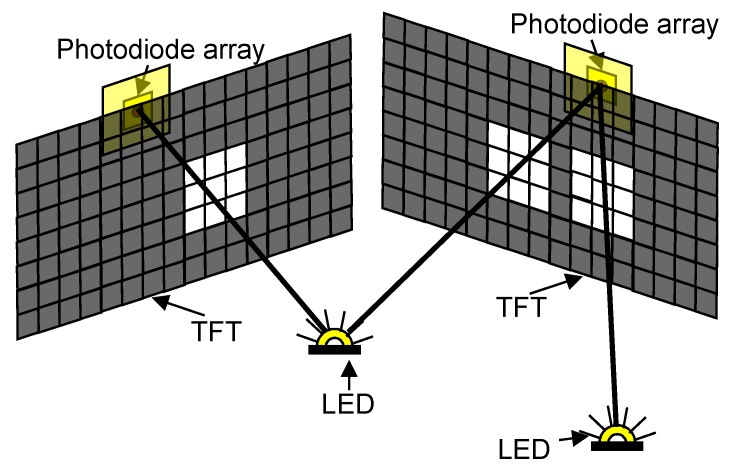
Theoretical scheme of the positioning system.

The position of a marker can be determined at least from two measurements made by sensors positioned far enough from each other. The sensor units are mounted to fixed positions so that each segment of the space of interest is in the field of vision of at least two units. Figure 5 shows the scheme of the positioning system.

The continuous operation requires the exact knowledge of the LEDs. Searching of the LEDs is required in two cases: For the initialization of the system and when the device looses the marker. Searching algorithms and the corresponding problems are out of the scope of this paper. The proposed device itself measures the angle of arrival, so in order to get 3D measurements, the principle of stereo-camera reconstruction should be used. This is discussed in detail in [16,17], so the main focus of this paper is on the measuring apparatus.

This positioning system has some potential advantages. It is well known [18] that a reliable link can be established between the LED marker and the sensing element, which makes this system very insensitive to ambient light, even if the ambient light changes rapidly. Such a situation can occur, e.g., in a manufacturing facility where arc welding takes place. Since the number of sensing elements is small, the computational requirements are negligible compared to image processing. Moreover, every part of the system is available on the market in mass production, so the price of the system is low.

## 3. Light Sensor

The main task of the light sensor is the recognition of the markers and the estimation of the intensity of their emitted light. This measurement is the basis of marker tracking, since the shadow of the patch should be detected in order to be able to track the marker. The light sensor measures the light intensity that hits the surface of the light detector. Therefore, the detector produces a continuous signal, which is sampled so that the result is a discrete signal. The presented prototype incorporates photodiodes as detectors. The light intensity estimation and the marker recognition is done by digital processing of the sampled signal. The technical details of the detector are not in the scope of this paper, but the simultaneous marker recognition and light intensity estimation are briefly discussed. The reliability of the connection of the light-emitting element and a detector is obvious; many industrial applications use a similar setup. For more about the opto-detector design, the reader is referred to [18].

Once the output signal of the photodiode is sampled, then the measured signal should be processed in a way that the independent markers and their light intensity can be estimated separately. There are several solutions for this problem. This paper presents a possible approach.

The basic idea is that the LEDs are flashing at different given frequencies. Exploiting that each LED blinks at a particular frequency, one can take the Fourier transformation of the signal. The frequency that belongs to the particular LED appears as a spike in the result of the transformation, and the absolute value of that point is related to the luminance of the particular LED. It is evident that the incoming signal is sampled so the applied transformation is the discrete Fourier transformation. Let $x_{n}$ be the incoming discrete signal. One can acquire *N* samples and take the discrete Fourier transformation on that window. Therefore, the luminance of a LED, which flashes at a predefined frequency, can be estimated with the following basic formula:

(1) $$I_{LED}=\left|\sum_{n=0}^{N-1}x_{n}e^{-j2\pi \frac{k}{N}n}\right|$$

where $I_{LED}$ is the estimated luminance and *k* is an integer that corresponds to the flashing frequency of the LED at the given sampling frequency. The estimation should be done for each LED separately. It is also obvious that the unit of $I_{LED}$ is irrelevant.

The measurement is performed as follows. The first step is to acquire *N* samples. Calculate $I_{LED}$, then start sampling again. In this setup, the light intensity is sampled $\frac{1}{N}$-times slower than the sample rate of the incoming discrete signal. The prototype uses a 1-ms time window and a 400-ksps sampling rate, and the blinking frequencies of the markers is 10 kHz, 11kHz, 12 kHz, *etc.* Therefore, the sampling frequency of the light intensity is 1 kHz.

The above outlined strategy can be further improved because the Fourier transformation works efficiently when the LED blinks at 50% duty cycle. The opto-coupler performs better if the LED emits short, but powerful pulses. In order to eliminate these concurring requirements, we can apply wavelet transformations [19]. Since the incoming signal is a sum of square waves, a possible solution is the Haar transformation. The wavelet transformation is computationally more expensive than Fourier transformation in general, but the dilatation parameter of a wavelet transformation is a predefined design parameter (*i.e.*, the flashing characteristic of the LEDs), so the difference is not significant from this point of view.

## 4. Modeling

It is essential to understand the governing dynamics of the TFT and LED combined structure. The modeling focuses on the dependency between the LED position, the patch position and the measurable light intensity of each photodiode. In order to make the discussion simpler, certain assumptions are made.

First, assume that the patch is rectangular. Second, consider that each wall of the patch can be moved independently. Therefore, for example, as the LED moves right (Figure 4), the photodiode on the right side becomes shadowed, and the photodiode on the left fully lights up. One can correct the position of the patch by moving the walls independently: The left wall should be moved to the right to shadow the left photodiode, and similarly, the right wall should be moved to light up the right photodiode. The walls do not need information about the others, because they can correct their position depending on the corresponding photodiode. This kind of tracking strategy significantly reduces the complexity of the system. Therefore, it is enough to examine the output of one photodiode and one wall pair. For the sake of simplicity, the bottom photodiode is chosen as the subject of examination. To further simplify the discussion, it is also assumed that only the bottom wall of the patch is moving and that the LED moves only along the vertical axis. This simplification means that only the rows of the TFT are controlled, and the columns remain unchanged.

The summary of the discussion is that the LED moves up and down while the bottom wall tracks its movement, and the model describes the correspondence between the LED position, the wall position (call it the patch position) and the light intensity of the LED.

**Figure 6:**
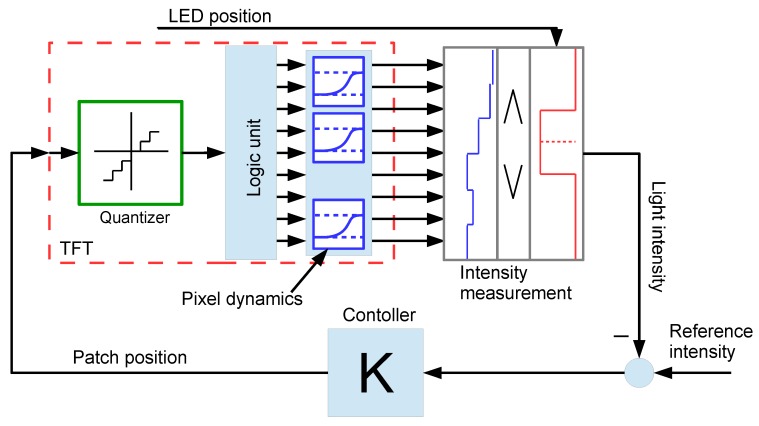
The LED tracking.

The model is constructed as a series of blocks. The inputs of the model are first the patch position, which is the coordinate of the wall of the patch corresponding to the examined photodiode (*i.e.*, the bottom photodiode), and second, the LED position that is considered as the intersection of the TFT screen and the line-of-sight. The LED position is measured in pixels, and fractions of pixels are also allowed. The output of the system is the measured light intensity of the LED. The output of a photodiode is a real number, and this value would be an input to the tracking controller, so it is practical to handle the patch position as a real number. The overall model can be seen in Figure 6.

The first sub-block in the TFT block is the quantizer. It is necessary because the patch position is modeled as a real value, so it has to be quantized according to the TFT resolution. The logic unit takes the quantized patch position and generates the input of each pixel. The pixels can be opaque or transparent, so the input of each pixel can be modeled as a binary value. The logic unit turns every pixel into a transparent state, which has a greater coordinate than the patch position; it is given as follows:

if the patch position >1, then drive the first pixel into the opaque state
if the patch position >2, then drive the second pixel into the opaque state
if the patch position >3, then drive the third pixel into the opaque state
.
.
.


The following block represents the TFT pixel dynamics. Once a pixel changes its state, the changes of the transparency are not immediate. It is common that the transparency response is different according to the direction of the change, that is, if the pixel turns into black from white, it is faster than the opposite direction. The transient response is modeled as a second order LTI (Linear Time Invariant) system. This simplification assumes that in the particular TFT unit, each direction has a similar transient response. Each output of the TFT dynamics block corresponds to the transparency of that particular row. The consecutive transparencies of the pixels along the vertical axis form a staircase function. This function plays an important role in calculating the light intensity of the LED.

In order to calculate the light intensity, the amount of light hitting the photodiode should be calculated. The transparency of the pixels between the LED and the photodiode plays a key role. Only the pixels above the photodiode are important, because the transparency of other pixels does not contribute to the measurable intensity. Thus, the surface of the photodiode can be projected to the TFT screen along the line-of-sight.

**Figure 7:**
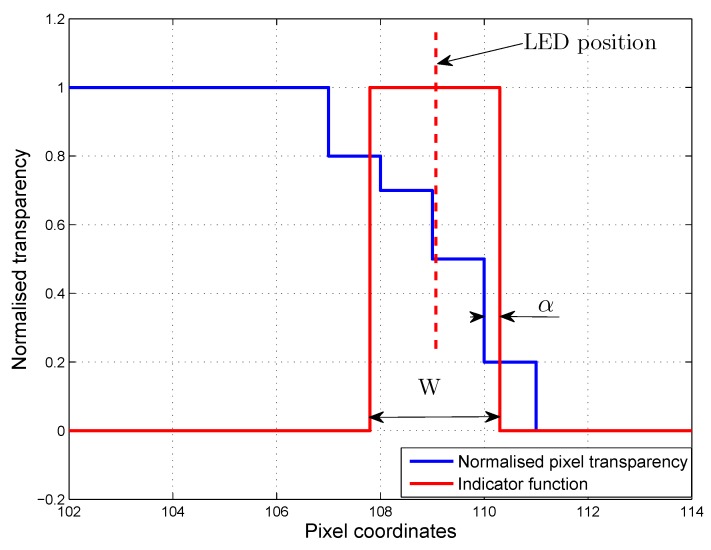
Light intensity measurement model.

The back projection can be interpreted as an indicator function. This indicator function is one if the photodiode is there and zero otherwise. It is easy to see that the measured light intensity is the dot product of the staircase function of the transparency and the indicator function. The middle of the nonzero part of the indicator function is always the LED position, since the back projection is done along the line of sight; see Figure 7.

Since the transparency function and the indicator function is piecewise constant, the dot product of the two functions is a summation. Therefore, let *x* be a variable along the vertical line, f(x) be the transparency function and g(x) be the indicator function, then the following formula is obtained:

(2) $$I_{LED}=\int_{0}^{K}f(x)g(x)dx=\sum_{0}^{K-1}f_{i}g_{i}=\gamma \left(L_{p}\right)\varphi$$

where $I_{LED}$ is the intensity, *K* is the coordinate of the last pixel, $f_{i}$ is the value of *f* corresponding to the *i*-th pixel and $g_{i}$ is the weight of the *i*-th pixel regarding the indicator function. If the indicator function is zero along the whole *i*-th pixel, the weight $g_{i}$ is zero; if the indicator function is one, the weight $g_{i}$ is one. If the indicator function partially covers a pixel, then $g_{i}=\int_{i}^{i+1}g(x)dx$. Of course, the summation can be rewritten in vector form where γ is the vector of $g_{i}$ and φ is the vector of $f_{i}$. The γ vector is a function of the LED position ($L_{p}$), while the φ vector is the function of the patch position and its past. Let the width of the non-zero part of the indicator function be *w*. In general, *w* is not a multiple of the pixel width, so the edge of the indicator function is shifted by *α* from the grid determined by pixels; see Figure 7. Using this notation, the γ vector can be expressed with *w* and *α*. As an example, let 2<w<3, and the LED position is at the 4th pixel. Then, the γ vector is:

$$\gamma =\left[\begin{array}{cccccccccc} 0 & 0 & w-\alpha -2 & 1 & 1 & \alpha & 0 & \cdots & 0 & 0 \end{array}\right]$$

and in general:

$$\gamma =\left[\begin{array}{cccccccccc} 0 & 0 & \cdots & w-\alpha -2 & 1 & 1 & \alpha & \cdots & 0 & 0 \end{array}\right].$$

The system can be described in a state space form from the output of the logic block to the light intensity output:

(3) $$\begin{array}{cc} \dot{x} & =\left[\begin{array}{cccc} A_{2\times 2} & 0 & \cdots & 0\\ 0 & A_{2\times 2} & \cdots & 0\\ \vdots & \vdots & \ddots & \vdots \\ 0 & 0 & \cdots & A_{2\times 2} \end{array}\right]x+\left[\begin{array}{cccc} B_{2\times 1} & 0 & \cdots & 0\\ 0 & B_{2\times 1} & \cdots & 0\\ \vdots & \vdots & \ddots & \vdots \\ 0 & 0 & \cdots & B_{2\times 1} \end{array}\right]u\\ y & =\gamma (L_{p})cx \end{array}$$

where *x* is the state vector, $A_{2\times 2}$ , $B_{2\times 1}$ are the system matrices of each pixel and *c* is the output matrix. The output of the system is the transparency of the pixels, so the values of *c* is straightforward, and *u* is the output of logic block in vector form.

The above discussed model describes the system along one dimension of the TFT, but the discussion can be extended to the whole surface, since the dot product can be calculated between surfaces, as well. The model discusses only one wall of the patch, but as the model is extended into two dimensions, the whole problem can be treated with this framework. The light interference is also treatable with this approach, since the interference can be included in the transparency function, but in practical cases, the interference is negligible. From a control design point of view, the presented model is still complicated, so further simplification is done.

## 5. Control Design

The controller is responsible for controlling the motion of the patch, such that it tracks the LED marker. The input of the controller is the estimated light intensity $I_{\mathrm{LED}}$, and the output is the patch position. Since the wall of the patch is controlled separately, each patch has four independent controller. Two control strategies are presented.

A simple control method can be obtained by applying the following two simple rules: If the light intensity is above a certain level, the patch position is moved so that the photodiode becomes shadowed, and if the light intensity is below a certain level, the patch position is moved in the opposite direction. This is a simple hysteresis control. Proper tuning of hysteresis solves the control problem, but better performance can be achieved with other control algorithms.

Since the tracking algorithms have to be implemented on microcontrollers with limited computation power, the goal of the design is to obtain a simple, but effective controller that performs sufficiently, even if light intensity measurement noises are taken into account. As a possible solution, a PID controller can be used. The control design is split into two phases. In the first step, the model is simplified, since the model of the marker tracking dynamics contains nonlinear elements. In order to design a PID, the nonlinear elements are either linearized or neglected. Then, the parameters of the PID controller are tuned.

The number of pixels and, thus, the order of the system, together with the nonlinearities, make the full model inconvenient for control design purposes. In order to obtain a baseline controller, the model of the marker tracking is simplified as follows.

- The quantization effect is neglected.
- One can choose an arbitrary operating point, and the control should operate around that point. Therefore, the logic block can be represented as a pass through around each operating point.
- The lightening and darkening dynamics of the TFT are different and, therefore, not suitable for classical control design techniques; for the sake of simplicity, they are approximated by a single transfer function as follows:
  (4) $$W_{TFT}=\frac{1}{T^{2}s^{2}+2\xi Ts+1}$$
  where *T* is the mean time constant and *ξ* is the damping ratio. Regarding the actual prototype T=8.75ms and ξ=0.9.
- The light intensity is a weighted sum of the outputs of each individual pixel. As the dynamics of every single pixel can be considered identical, an approximated dynamic model of the TFT matrix may be obtained by summing the transfer functions of pixels.

As a result, a digital controller operating at 50Hz can be designed. The main objectives of the control design are the short settling time and overshoot-free operation. A PI controller with the following parameters (standard form, *i.e.*, *P* acts on the integral term) turns out to be suitable for the control problem:

(5) P=1andI=50

**Figure 8:**
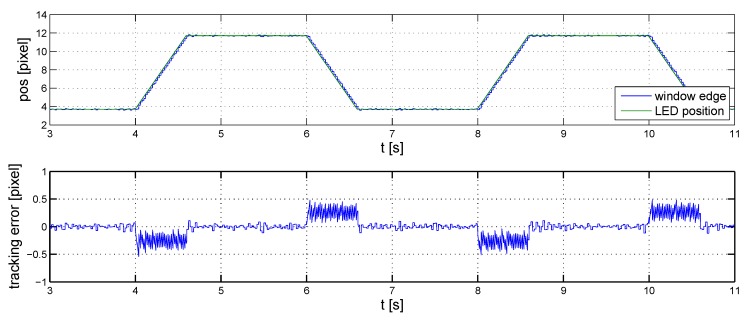
Initial controller, linear simulations.

The results of the initial linear simulations are shown in Figure 8, in which the position of the LED and that of the transparent window’s edge are marked red and blue, respectively. The measurements are loaded with a Gaussian noise with a standard deviation of one pixel. The speed of the marker in the simulation corresponds to that of a typical vehicle’s in indoor environments.

In the second step, the controller is tuned so that the performance of the controller connected to the full model remains satisfactory. Since the performance of the baseline controller deteriorates as the simplified model is replaced by the detailed one, the controller needs to be fine tuned. Due to the effect of quantization, the best result that may be achieved is that the transparent window oscillates about the real position of the marker, since it is not an integer value in general. The goal is then to minimize the amplitude of this oscillation.

**Figure 9:**
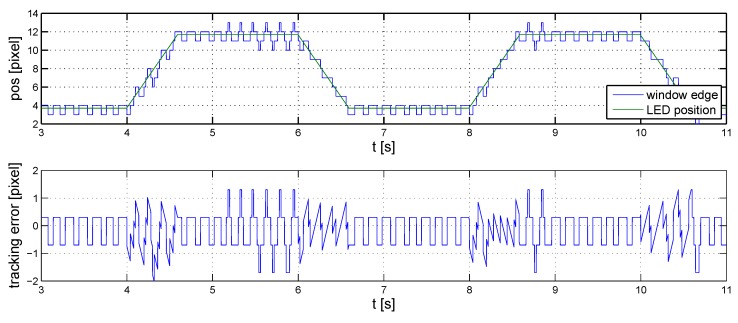
Initial controller, nonlinear simulations.

The baseline controller performs satisfactorily when the marker is in motion. However, it causes a continuous oscillation about stationary values with an amplitude of two pixels (Figure 9).

This oscillation can be reduced by introducing a derivative effect in the controller. The tuned parameters of the controller are:

(6) P=1,I=45andD=0.01

**Figure 10:**
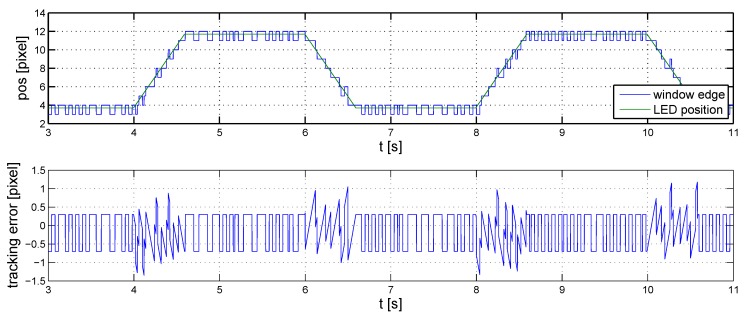
Fine-tuned controller, nonlinear simulations.

The performance of the controller is illustrated in Figure 10, where noise and reference signal settings are similar to those in the previous case. The improvement on the baseline controller is clearly visible, both when the LED is in motion and when it is stationary.

The controller is implemented on each wall of the patch independently, so the patch forms a rectangle, and the LED angle of arrival can be estimated with the center of this rectangle. The performance of the controller has a direct impact on the precision, so a more sophisticated control algorithm could perform better. The design and implementation of other control algorithms are part of the future work.

## 6. Prototype

**Figure 11:**
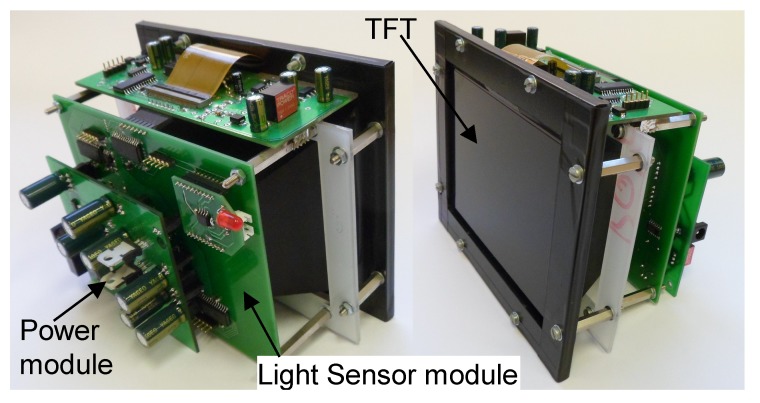
The prototype of the indoor positioning sensor.

To prove the concept, a prototype positioning system was built. The main element of the system, the sensor unit, can be seen in Figure 11.

**Figure 12:**
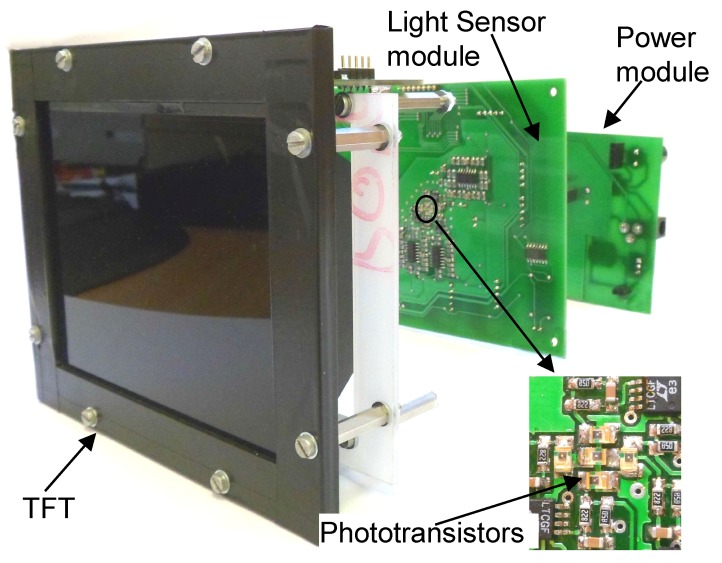
Explode view of the prototype.

The sensor unit consists of three main parts: The TFT and its driving circuits, the light sensor module, which contains the photodiodes, and the power supply module. The explode view (Figure 12) of the positioning sensor reveals the realized construction of the above-mentioned theoretical scheme.

The photodiode array is placed on the optical axis of the instrument; it can be seen in the middle of the light sensor module surrounded by the analogue amplifier circuits.

The performance of the prototype is demonstrated in two test scenarios. During the first test, a randomly-placed LED of a fixed position is measured by the instrument. The test focuses on the developed control algorithms.

**Figure 13:**
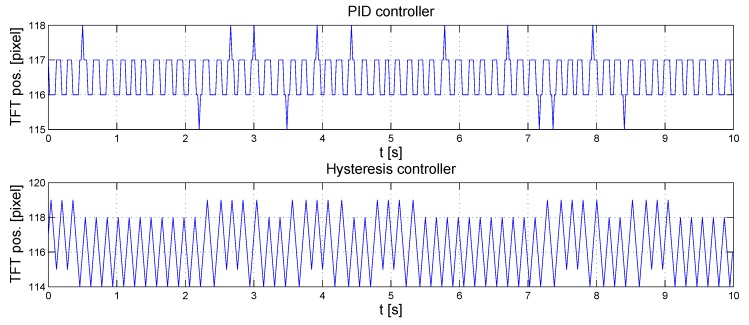
Comparison of hysteresis and PID controller.

The hysteresis controller and the PID controller are compared in Figure 13. According to the diagram, the measured deflection is one pixel most of the time with the PID controller. Hence, a precision of one pixel is reachable. The hysteresis controller performs worse because it neglects the dynamics of the pixels.

**Figure 14:**
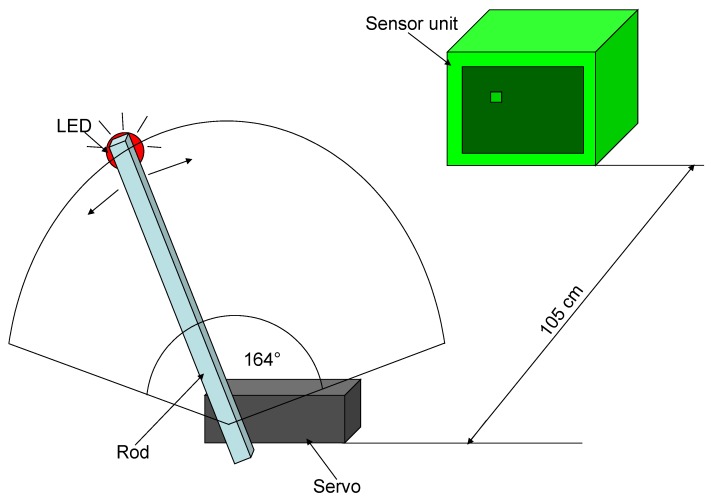
Tracking test setup.

The marker tracking capability is tested with the second test setup. These results can be found in [15], as well. The marker is attached to a stick, moving on a predefined route (as shown in Figure 14). The stick is swung by a servo motor, thus the marker stays on a fixed route while moving.

**Figure 15:**
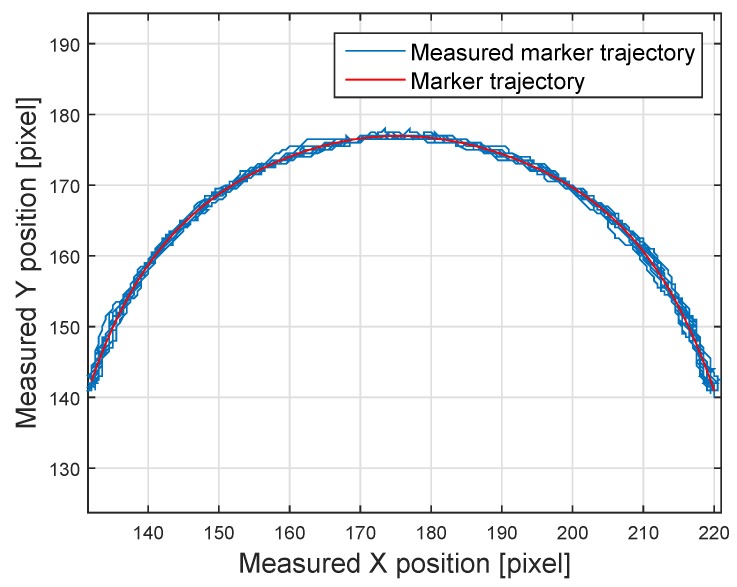
Measured *vs.* theoretic moving marker track.

**Figure 16:**
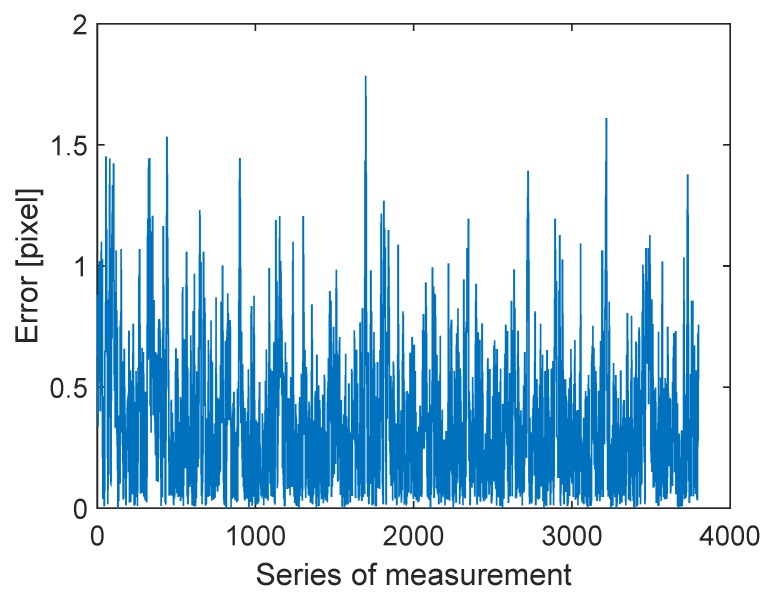
Measurement error.

The servo moves 164 degrees in both directions with a 15.2-s period. The stick’s length is 27.5 cm. The measurements of the LED coordinates are compared with the reference marker trajectory, illustrated in Figure 15. The error between the points and the theoretic curve remains about one pixel for most of the cases (see Figure 16).

The sensor unit has a 320 × 240 pixel resolution TFT panel produced by Display Elektronik GmbH, Part Number DEM320240H TMH-PW-N. The applied photodiodes are Model TEMD7000X01 by Vishay. Each signal of the photodiodes is processed by a dsPIC33FJ128GP802 microcontroller, and the LED tracking runs on the same type. The sensor unit has a 50-Hz refresh rate and a 90-degree viewing angle, making it capable of tracking an object that moves 14.06 degrees per second if the patch is moved by one pixel per frame. The device can track an object traveling by 0.73 m/s from a three-meter distance. The tracking speed can be increased at the expense of resolution. The main limitation in the prototype is the response time, which is about 25 ms. The most advanced TFT panels have a response time of less than 2 ms, so the traceable speed is satisfactory for the indoor positioning of mobile robots or to capture human motion. The problem with the faster TFT panels is that their fast response time cannot be fully exploited, as the refresh rate of the current TFT panels is not optimized for high frequency operation. This limitation is a matter of the TFT driver rather than that of the TFT technology.

The prototype consists of four sensor units installed on the top corners of a room of a size of four by four meters. The mean of the measured traceable speed was 0.6 m/s, which is slightly below the reachable maximum. The standard deviation of the position is about 0.9 cm.

The proposed prototype has been applied in a coaxial helicopter experimental platform; more about this application is shown in [15].

## 7. Conclusions

A original conceptual optical indoor positioning technique has been outlined. The marker recognition is realized with optical detectors, which deliver insensitivity to ambient light. The tracking is solved based on the simple principles of the pinhole camera. A proof-of-concept prototype is built to analyze the feasibility of the system and led to promising results.

The main advantage of this solution is that the marker recognition is solved in a very robust and reliable way. The simplicity of the system makes it very inexpensive and computationally less demanding than camera-based systems.

In the future, the improvement of the tracking controller and a solution for a more sophisticated light intensity measurement can give a solid basis to further development.
